# Association of Concomitant Bone Resorption Inhibitors With Overall Survival Among Patients With Metastatic Castration-Resistant Prostate Cancer and Bone Metastases Receiving Abiraterone Acetate With Prednisone as First-Line Therapy

**DOI:** 10.1001/jamanetworkopen.2021.16536

**Published:** 2021-07-22

**Authors:** Edoardo Francini, Francesco Montagnani, Pier Vitale Nuzzo, Miguel Gonzalez-Velez, Nimira S. Alimohamed, Pietro Rosellini, Irene Moreno-Candilejo, Antonio Cigliola, Jaime Rubio-Perez, Francesca Crivelli, Grace K. Shaw, Li Zhang, Roberto Petrioli, Carmelo Bengala, Guido Francini, Jesus Garcia-Foncillas, Christopher J. Sweeney, Celestia S. Higano, Alan H. Bryce, Lauren C. Harshman, Richard Lee-Ying, Daniel Y. C. Heng

**Affiliations:** 1 Department of Experimental and Clinical Medicine, University of Florence, Florence, Italy; 2 Department of Oncology, Ospedale degli Infermi, Biella, Italy; 3 Lank Center for Genitourinary Oncology, Dana Farber Cancer Institute, Harvard Medical School, Boston, Massachusetts; 4 Genomic Oncology Clinic, Mayo Clinic, Phoenix, Arizona; 5 Division of Medical Oncology, Tom Baker Cancer Centre, Calgary, Alberta, Canada; 6 Department of Medical and Surgical Sciences and Neuroscience, University of Siena, Siena, Italy; 7 Medical Oncology Unit, Hospital HM Sanchinarro Start, Madrid, Spain; 8 University Hospital Fundacion Jimenez Diaz, Autonomous University of Madrid, Madrid, Spain; 9 DFCI at Geisinger Medical Center, Danville, Pennsylvania; 10 Medical Oncology Unit, Misericordia Hospital, Grosseto, Italy; 11 Fred Hutchinson Cancer Research Center, University of Washington, Seattle

## Abstract

**Importance:**

Bone resorption inhibitors (BRIs) are recommended by international guidelines to prevent skeletal-related events (SREs) among patients with metastatic castration-resistant prostate cancer (mCRPC) and bone metastases. Abiraterone acetate with prednisone is currently the most common first-line therapy for the treatment of patients with mCRPC; however, the clinical impact of the addition of BRIs to abiraterone acetate with prednisone in this disease setting is unknown.

**Objective:**

To evaluate the association of the use of concomitant BRIs with overall survival (OS) and time to first SRE among patients with mCRPC and bone metastases receiving abiraterone acetate with prednisone as first-line therapy.

**Design, Setting, and Participants:**

This retrospective cohort study collected data from 745 consecutive patients who began receiving abiraterone acetate with prednisone as first-line therapy for mCRPC with bone metastases between January 1, 2013, and December 31, 2016. Data were collected from 8 hospitals in Canada, Europe, and the US from June 15 to September 15, 2019.

**Exposures:**

Patients were classified by receipt vs nonreceipt of concomitant BRIs and subclassified by volume of disease (high volume or low volume, using definitions from the Chemohormonal Therapy Vs Androgen Ablation Randomized Trial for Extensive Disease in Prostate Cancer [CHAARTED] E3805 study) at the initiation of abiraterone acetate with prednisone therapy.

**Main Outcomes and Measures:**

The primary end point was OS. The secondary end point was time to first SRE. The Kaplan-Meier method and Cox proportional hazards models were used.

**Results:**

Of the 745 men (median age, 77.6 years [interquartile range, 68.1-83.6 years]; 699 White individuals [93.8%]) included in the analysis, 529 men (71.0%) received abiraterone acetate with prednisone alone (abiraterone acetate cohort), and 216 men (29.0%) received abiraterone acetate with prednisone plus BRIs (BRI cohort). A total of 420 men (56.4%) had high-volume disease, and 276 men (37.0%) had low-volume disease. The median follow-up was 23.5 months (95% CI, 19.8-24.9 months). Patients in the BRI cohort experienced significantly longer OS compared with those in the abiraterone acetate cohort (31.8 vs 23.0 months; hazard ratio [HR], 0.65; 95% CI, 0.54-0.79; *P* < .001). The OS benefit in the BRI cohort was greater for patients with high-volume vs low-volume disease (33.6 vs 19.7 months; HR, 0.51; 95% CI, 0.38-0.68; *P* < .001). The BRI cohort also had a significantly shorter time to first SRE compared with the abiraterone acetate cohort (32.4 vs 42.7 months; HR, 1.27; 95% CI, 1.00-1.60; *P* = .04), and the risk of a first SRE was more than double in the subgroup with low-volume disease (HR, 2.29; 95% CI, 1.57-3.35; *P* < .001). In the multivariable analysis, concomitant BRIs use was independently associated with longer OS (HR, 0.64; 95% CI, 0.52-0.79; *P* < .001).

**Conclusions and Relevance:**

In this study, the addition of BRIs to abiraterone acetate with prednisone as first-line therapy for the treatment of patients with mCRPC and bone metastases was associated with longer OS, particularly in patients with high-volume disease. These results suggest that the use of BRIs in combination with abiraterone acetate with prednisone as first-line therapy for the treatment of mCRPC with bone metastases could be beneficial.

## Key Points

**Question:**

Do patients receiving abiraterone acetate with prednisone as first-line therapy for the treatment of metastatic castration-resistant prostate cancer with bone metastases benefit from the addition of bone resorption inhibitors?

**Findings:**

In this cohort study of 745 patients receiving first-line abiraterone acetate with prednisone for the treatment of metastatic castration-resistant prostate cancer with bone metastases, the use of concomitant bone resorption inhibitors was associated with improvements in overall survival, particularly among those with a high volume of disease.

**Meaning:**

These findings suggest that the addition of bone resorption inhibitors to abiraterone acetate with prednisone as first-line therapy could be beneficial for the treatment of patients with metastatic castration-resistant prostate cancer with bone metastases.

## Teaser

This cohort study examines whether the addition of bone resorption inhibitors is associated with improvements in overall survival and skeletal-related events among patients with metastatic castration-resistant prostate cancer receiving abiraterone acetate with prednisone as first-line therapy.

## Introduction

Prostate cancer is associated with the death of more than 360 000 men per year worldwide.^1^ Metastatic castration-resistant prostate cancer (mCRPC) is the advanced state of disease during which most deaths occur, and the median overall survival (OS) among patients with mCRPC is approximately 30 months.^2,3,4^ Mortality risk increases with the spread of the disease to the bone, which is a preferential site of metastasis for prostate cancer.^5^ In this respect, the number of bone metastases, the presence of bone pain, and the occurrence of skeletal-related events (SREs) is associated with shorter survival.^6,7^

Zoledronic acid, the most potent of the bisphosphonates, and denosumab, a monoclonal antibody directed against the receptor activator of nuclear factor κB ligand (RANKL), are osteoclast-mediated bone resorption inhibitors (BRIs) that are approved for the prevention of SREs in patients with CRPC and bone metastases and are recommended by international guidelines for use in this disease setting.^8,9,10,11,12^ However, neither of these agents alone was associated with a survival benefit among men with CRPC and bone metastases in phase 3 randomized clinical trials.^13^ Nevertheless, post hoc analyses of prospective studies suggested that the combination of BRIs with the androgen biosynthesis inhibitor abiraterone acetate with prednisone or the bone-targeting radionuclide radium-223 may provide a survival benefit compared with placebo.^14,15^ In this regard, receipt of abiraterone acetate with prednisone vs placebo with prednisone was associated with increases in the longevity of chemotherapy-naive patients with mCRPC in the pivotal phase 3 study, COU-AA-302 (Abiraterone Acetate Plus Prednisone Vs Placebo Plus Prednisone in Chemotherapy-Naïve Men With Metastatic Castration-Resistant Prostate Cancer).^3^ In addition, abiraterone acetate with prednisone was associated with significant reductions in pain, delays in pain progression, and, similar to zoledronic acid and denosumab, prevention of SREs.^16^

Abiraterone acetate with prednisone is currently the most commonly administered first-line therapy for patients with mCRPC.^17^ Because BRIs do not impact OS and because abiraterone acetate with prednisone has been associated with the prevention of SREs, the addition of BRIs is often omitted based on the rationale that BRIs would not add benefit to abiraterone acetate with prednisone alone. Nonetheless, BRIs and abiraterone acetate with prednisone have different and potentially complementary mechanisms of action; thus, it is possible that their concomitant use may produce synergistic benefits.^8,10,18^ However, to date, the clinical outcomes of the addition of BRIs to abiraterone acetate with prednisone compared with abiraterone acetate with prednisone alone as first-line therapy for patients with mCRPC and bone metastases have not been investigated. Given the potential for adverse events, including hypocalcemia, osteonecrosis of the jaw, and kidney impairment, and the costs associated with BRI use, evaluating whether these agents add clinical benefit to abiraterone acetate with prednisone is an important clinical question.^8,9,10^

In this study, we sought to assess the clinical impact associated with BRIs among men with mCRPC and bone metastases receiving abiraterone acetate with prednisone as first-line therapy. We also conducted a subgroup analysis to evaluate the clinical outcomes of the combination of abiraterone acetate with prednisone plus BRIs vs abiraterone acetate with prednisone alone among patients with high-volume vs low-volume disease.^19^

## Methods

This cohort study followed the Strengthening the Reporting of Observational Studies in Epidemiology (STROBE) reporting guideline. Eight institutions in Canada, Europe, and the US participated in the study, and institutional review board approval was granted in each center before the study began. The study was given a waiver of informed consent because all data were deidentified.

### Study Cohorts

Clinical and electronic registries from the 8 participating hospitals were queried to identify a cohort of consecutive patients with histologically confirmed CRPC (defined using Prostate Cancer Clinical Trial Working Group 3 criteria^20^) and radiologically quantifiable bone metastases who began receiving abiraterone acetate with prednisone as first-line therapy for mCRPC between January 1, 2013, and December 31, 2016. This specific time frame accounted for the European Medicines Agency approval date of abiraterone acetate with prednisone for the treatment of chemotherapy-naive mCRPC (2013) and the need for an adequate follow-up period. Data were collected from June 15 to September 15, 2019.

Patients who had received any therapy for mCRPC before initiation of abiraterone acetate with prednisone and patients who had received abiraterone acetate with prednisone for the treatment of metastatic hormone-sensitive prostate cancer were not included in the study. Patients who experienced an SRE (defined as palliative local therapy to bone, pathological fractures, or spinal cord compression) before beginning treatment with abiraterone acetate with prednisone were excluded, as were those who initiated treatment with zoledronic acid or denosumab therapy more than 3 months after or before the initiation of abiraterone acetate with prednisone, with the exception of osteoporosis treatment.

Demographic, pathologic, and clinical data, including type of metastatic disease presentation (de novo or recurrent after previous local therapy), treatment for metastatic hormone-sensitive prostate cancer (androgen deprivation therapy or androgen deprivation therapy plus docetaxel), volume of disease (high or low), presence of any visceral metastases (including liver), presence of liver metastases, Eastern Cooperative Oncology Group (ECOG) performance status, intensity of cancer-associated pain (hereinafter referred to as pain), receipt of opioids at baseline, date of initiation of palliative therapy for pain intensity, prostate-specific antigen measurements, and all information related to BRI and abiraterone acetate with prednisone treatments were collected from medical and electronic records. All patients were followed up until death, last follow-up visit, or September 15, 2019 (data cutoff date), whichever occurred first.

Patients were categorized into 2 cohorts based on the receipt or nonreceipt of concomitant BRIs; the abiraterone acetate cohort received abiraterone acetate with prednisone alone, and the BRI cohort received abiraterone acetate with prednisone plus BRIs. Patients were also subclassified by volume of disease, with high-volume disease defined as visceral metastases and/or at least 4 bone metastases, including 1 or more metastases out of the axis and pelvis, and low-volume disease defined as the absence of high-volume disease (based on definitions from the Chemohormonal Therapy Vs Androgen Ablation Randomized Trial for Extensive Disease in Prostate Cancer [CHAARTED] E3805 study^19^).

### Outcomes

The primary end point of the study was OS, defined as the time from initiation of treatment with abiraterone acetate with prednisone to the date of death from any cause, the date of the last recorded clinical appointment, or the date of data cutoff, whichever came first. The secondary end point was time to first SRE, defined as the time from the initiation of abiraterone acetate with prednisone to the date of the first radiologically documented SRE, the date of the last follow-up visit, the date of data cutoff, or the date of death from any cause, whichever occurred first.

### Statistical Analysis

Median values with interquartile ranges (IQRs) and frequencies with percentages were calculated for the description of continuous and categorical variables, respectively. Medians and proportions were compared using unpaired 2-sided *t* tests and χ^2^ tests, respectively. The Kaplan-Meier method was used to calculate the distributions of the end points, including median time to event with 95% CIs, and a log-rank test was applied to compare time to event distributions among the cohorts. The Cox proportional hazards model was used to compare the estimates of the outcomes among the groups.

All variables were first tested using a univariable analysis. Variables with significant association with the outcomes were used to build a multivariable model using the stepwise method, after considering collinearity among variables using a correlation matrix with a cutoff value of 0.7. Variables higher than this threshold were further tested using the variance inflation factor. In cases in which the variance inflation factor was greater than 5, the collinearity was deemed excessive, and modifications to the model were considered, including the removal of the variable. In our database, less than 10% of patients had some missing data (at random); therefore, complete case analyses were performed to remove patients with missing data for the variable of interest. To address the potential bias associated with the exclusion of missing data, we compared characteristics of patients included in the multivariable model for OS (without missing data) with those of the general population (eTable 1 in the Supplement).

The assumption of proportionality was verified by plotting log-minus-log survival curves and by cumulative martingale process plots. First-order interactions were assessed for each multivariable model. Covariates included in the multivariable Cox models were clinicopathological factors known to be associated with survival and time to first SRE, such as Gleason score, previous local therapy (surgery or radiotherapy), de novo disease, receipt of treatment for metastatic hormone-sensitive prostate cancer, baseline volume of disease, liver metastases, ECOG performance status, pain intensity, receipt of opioids, and prostate-specific antigen value.^7,21,22,23^

All statistical tests were 2-sided with a significance threshold of *P* ≤ .05. Statistical analyses were performed using SAS software, version 9.4 (SAS Institute), and R software, version 3.4.0 (R Foundation).

## Results

### Patient Characteristics

Of the 745 patients (median age, 77.6 years [IQR, 68.1-83.6 years]; 699 White individuals [93.8%]) eligible for this analysis, 529 patients (71.0%) received abiraterone acetate with prednisone alone, and 216 patients (29.0%) received abiraterone acetate with prednisone plus concurrent BRIs (eFigure in the Supplement). Demographic and pathologic characteristics were comparable between the 2 cohorts (Table 1). A total of 420 men (56.4%) had high-volume disease, and 276 men (37.0%) had low-volume disease. The median follow-up was 23.5 months (95% CI, 19.8-24.9 months). In the BRI cohort, 132 patients (61.1%) received denosumab, and 84 patients (38.9%) received zoledronic acid. The median time from the initiation of treatment with abiraterone acetate with prednisone to the addition of BRIs was 15 days (IQR, 0-68 days), and the median duration of BRI use was approximately 2 years (median, 741 days; IQR, 362-1182 days).

**Table 1.** Patient Characteristics

Characteristic	No. (%)			*P* value
	Overall population (N = 745)	Abiraterone acetate cohort (n = 529)	BRI cohort (n = 216)	
Age at baseline, median (IQR), y	77.6 (68.1-83.6)	78.3 (70.5-85.4)	75.8 (66.9-81.2)	.10
Race				
White	699 (93.8)	497 (94.0)	202 (93.5)	.08
Other^a^	19 (2.6)	10 (1.9)	9 (4.2)	
Not available	27 (3.6)	22 (4.2)	5 (2.3)	
Gleason score				
≤6	62 (8.3)	42 (7.9)	20 (9.3)	.53
7	218 (29.3)	145 (27.4)	73 (33.8)	
≥8	337 (45.2)	234 (44.2)	103 (47.7)	
Not available	128 (17.2)	108 (20.4)	20 (9.3)	
Previous local therapy				
No	353 (47.4)	265 (50.1)	88 (40.7)	.03
Surgery	196 (26.3)	127 (24.0)	69 (31.9)	
Radiotherapy	181 (24.3)	125 (23.6)	56 (25.9)	
Not available	15 (2.0)	12 (2.3)	3 (1.4)	
De novo				
No	483 (64.8)	340 (64.3)	143 (66.2)	.64
Yes	262 (35.2)	189 (35.7)	73 (33.8)	
Treatment for mHSPC				
ADT	617 (82.8)	475 (89.8)	142 (65.7)	<.001
ADT plus docetaxel	81 (10.9)	41 (7.8)	40 (18.5)	
Not available	47 (6.3)	13 (2.5)	34 (15.7)	
Volume of disease at baseline^b^				
Low	276 (37.0)	179 (33.8)	97 (44.9)	<.001
High	420 (56.4)	336 (63.5)	84 (38.9)	
Not available	49 (6.6)	14 (2.6)	35 (16.2)	
Visceral metastases at baseline				
No	680 (91.3)	477 (90.2)	203 (94.0)	.09
Yes	65 (8.7)	52 (9.8)	13 (6.0)	
Liver metastases at baseline				
No	709 (95.2)	506 (95.7)	203 (94.0)	.33
Yes	36 (4.8)	23 (4.3)	13 (6.0)	
ECOG performance status at baseline				
0	324 (43.5)	224 (42.3)	100 (46.3)	.01
≥1	369 (49.5)	287 (54.3)	82 (38.0)	
Not available	52 (7.0)	18 (3.4)	34 (15.7)	
Pain intensity at baseline, VAS				
0-3	514 (69.0)	389 (73.5)	125 (57.9)	.03
4-5	112 (15.0)	71 (13.4)	41 (19.0)	
>5	70 (9.4)	53 (10.0)	17 (7.9)	
Not available	49 (6.6)	16 (3.0)	33 (15.3)	
Receiving opioids at baseline				
No	598 (80.3)	449 (84.9)	149 (69.0)	.05
Yes	99 (13.3)	65 (12.3)	34 (15.7)	
Not available	48 (6.4)	15 (2.8)	33 (15.3)	
PSA at baseline, median (IQR), ng/mL	32 (10.0-98.5)	37.5 (12.2-126.2)	19.3 (6.4-57.0)	.18
Receiving a BRI				
No	529 (71.0)	529 (100)	0	NA
Zoledronic acid	84 (11.3)	0	84 (38.9)	
Denosumab	132 (17.7)	0	132 (61.1)	
Time from start of abiraterone acetate with prednisone to start of BRI, median (IQR), d	15 (0-68)	NA	15 (0-68)	NA
Duration of BRI receipt, median (IQR), d	741 (362-1182)	NA	741 (362-1182)	NA
No. of abiraterone acetate with prednisone cycles, median (IQR)	9 (4-20)	10 (4-21)	7 (4-14)	.01
Receiving pain palliation				
No	198 (26.6)	127 (24.0)	71 (32.9)	.002
Yes	497 (66.7)	386 (73.0)	111 (51.4)	
Not available	50 (6.7)	16 (3.0)	34 (15.7)	
No. of treatments received after abiraterone acetate with prednisone				
Median (IQR)	1 (0-2)	0 (0-1)	2 (1-3)	<.001
0-1	511 (68.6)	417 (78.8)	94 (43.5)	
>1	225 (30.2)	112 (21.2)	113 (52.3)	
Not available	9 (1.2)	0	9 (4.2)	
Follow-up in overall population, median (95% CI), mo	23.5 (19.8-24.9)	21.6 (17.2-25.3)	28.6 (22.5-34.3)	.36

Abbreviations: ADT, androgen deprivation therapy; BRI, bone resorption inhibitor; ECOG, Eastern Cooperative Oncology Group; IQR, interquartile range; mHSPC, metastatic hormone-sensitive prostate cancer; NA, not available/not applicable; PSA, prostate-specific antigen; VAS, visual analog scale.

^a^ Races included in this category were not specifically identified.

^b^ High-volume disease was defined as visceral metastases and/or at least 4 bone metastases, including 1 or more metastases out of the axis and pelvis. Low-volume disease was defined as the absence of high-volume disease.

As expected, at baseline, the proportion of patients with low pain intensity (defined as a score of <4 on the visual analog scale) was greater (389 patients [73.5%] vs 125 patients [57.9%]), and the receipt of opioids was less common (65 patients [12.3%] vs 34 patients [15.5%]) in the abiraterone acetate cohort vs the BRI cohort. High-volume disease was present in 336 patients (63.5%) in the abiraterone acetate cohort vs 84 patients (38.9%) in the BRI cohort. A total of 287 patients (54.3%) in the abiraterone acetate cohort vs 82 patients (38.0%) in the BRI cohort had an ECOG performance status of 1 or higher. The number of patients receiving fewer than 2 therapies for mCRPC after receipt of abiraterone acetate with prednisone was 417 (78.8%) in the abiraterone acetate cohort vs 94 (43.5%) in the BRI cohort. The rates of visceral metastases and liver metastases were relatively low and comparable between those who received abiraterone acetate with prednisone (52 patients [9.8%] and 23 patients [4.3%], respectively) and those who received abiraterone acetate with prednisone plus BRIs (13 patients [6.0%] and 13 patients [6.0%], respectively).

### Outcomes Associated With Concomitant BRI vs No BRI

**Figure 1.**
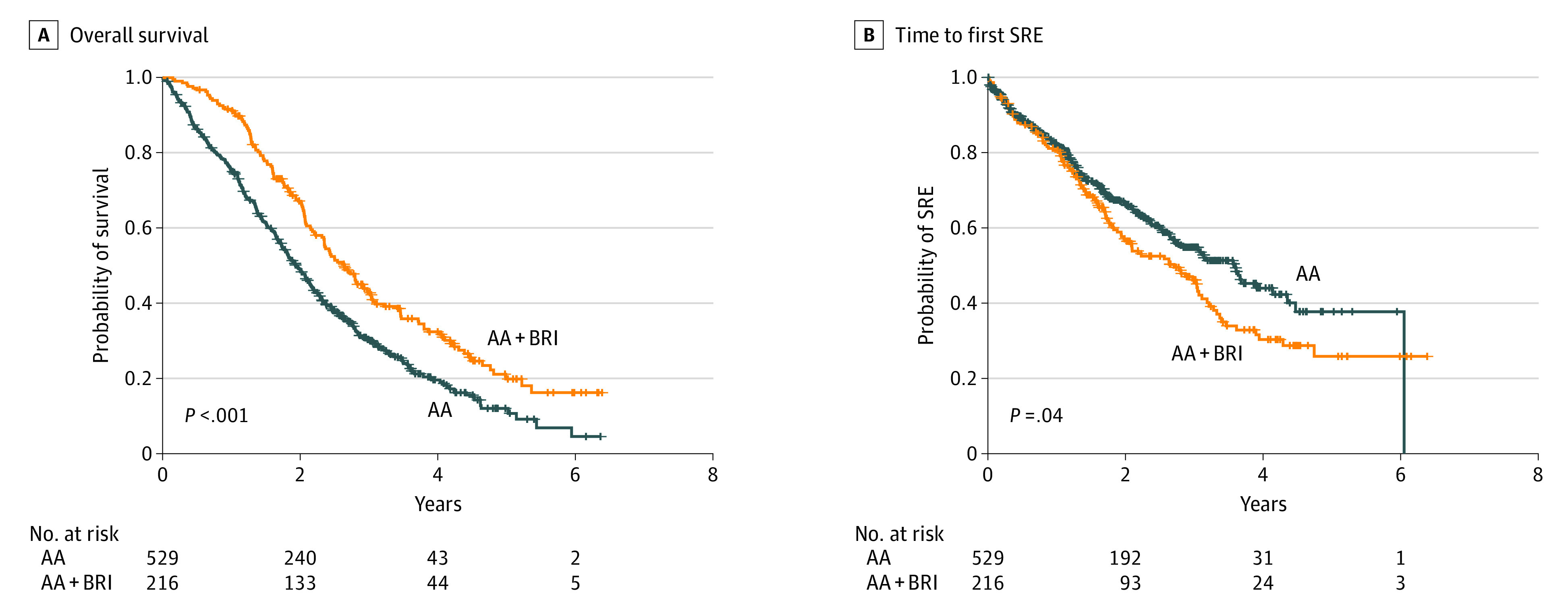
Overall Survival and Time to First Skeletal-Related Event (SRE) in Overall Population by Receipt of Bone Resorption Inhibitors An SRE was defined as palliative surgery or radiotherapy to bone, pathological fractures, or spinal cord compression. AA indicates abiraterone acetate plus prednisone; BRI, bone resorption inhibitor.

The median OS among the 216 patients in the BRI cohort was 31.8 months (95% CI, 28.2-36.4 months) compared with 23.0 months (95% CI, 21.0-25.7 months) among those in the abiraterone acetate cohort. The risk of death in the BRI cohort decreased by 35% (hazard ratio [HR], 0.65; 95% CI, 0.54-0.79; *P* < .001) (Table 2). The Kaplan-Meier estimates illustrating the survival benefit associated with the addition of BRIs to abiraterone acetate with prednisone therapy are shown in Figure 1A. In contrast, albeit with less statistical significance, the BRI cohort experienced a decrease in the median time to first SRE compared with the abiraterone acetate cohort (32.4 vs 42.7 months, respectively; HR, 1.27; 95% CI, 1.00-1.60; *P* = .04). The negative association of the receipt of concomitant BRIs with time to first SRE is shown in the Kaplan-Meier curves in Figure 1B.

**Table 2.** Clinical Outcomes Among Overall Population by Receipt of Bone Resorption Inhibitors

Outcome	Abiraterone acetate cohort (n = 529)	BRI cohort (n = 216)	HR (95% CI)	*P* value
Deaths, No.	369	147	NA	NA
OS, median (95% CI), mo	23.0 (21.0-25.7)	31.8 (28.2-36.4)	0.65 (0.54-0.79)	<.001
SREs, No.	188	116	NA	NA
Time to first SRE, median (95% CI), mo	42.7 (33.2-52.2)	32.4 (24.1-37.3)	1.27 (1.0-1.60)	.04

Abbreviations: BRI, bone resorption inhibitor; HR, hazard ratio; NA, not applicable; OS, overall survival; SRE, skeletal-related event.

In the multivariable analysis of OS, the addition of BRIs was independently associated with improvement in OS (HR, 0.64; 95% CI, 0.52-0.79; *P* < .001), which was consistent with the outcomes of the univariable model (HR, 0.65; 95% CI, 0.54-0.79; *P* < .001) (eTable 2 in the Supplement). The type of BRI (denosumab vs zoledronic acid) received was not associated with OS (HR, 0.93; 95% CI, 0.66-1.30; *P* = .79). Independent associations with increased OS were also found for low-volume vs high-volume disease (HR, 0.66; 95% CI, 0.54-0.80; *P* < .001), previous local therapy vs no previous local therapy (surgery vs no surgery: HR, 0.51; 95% CI, 0.39-0.68; *P* < .001; radiotherapy vs no radiotherapy: HR, 0.71; 95% CI, 0.55-0.93; *P* = .03), liver metastases vs no liver metastases (HR, 2.04; 95% CI, 1.37-3.05; *P* = .006), ECOG performance status of 0 vs 1 or higher (HR, 1.46; 95% CI, 1.20-1.78; *P* = .003), baseline pain of 3 or lower vs 5 or higher on the visual analog scale (HR, 1.88; 95% CI, 1.39-2.54; *P* < .001), and baseline prostate-specific antigen level (HR, 1.03; 95% CI, 1.01-1.05; *P* = .006). In the multivariable analysis of time to first SRE, compared with the results of the univariable model, no association was found between the receipt of concomitant BRIs vs no BRIs and delayed time to first SRE (eTable 3 in the Supplement). However, low-volume vs high-volume disease and baseline pain of 3 or lower vs 4 to 5 or higher than 5 on the visual analog scale revealed independent associations with improved time to first SRE. The survival advantage of concomitant BRI in the overall population remained significant when stratified by the covariates (eTable 4 in the Supplement) or centers (eTable 5 in the Supplement), even if a certain variability in covariate rates and overall survival data was observed across centers (eTable 6 in the Supplement).

### Subgroup Analysis by Disease Volume

**Figure 2.**
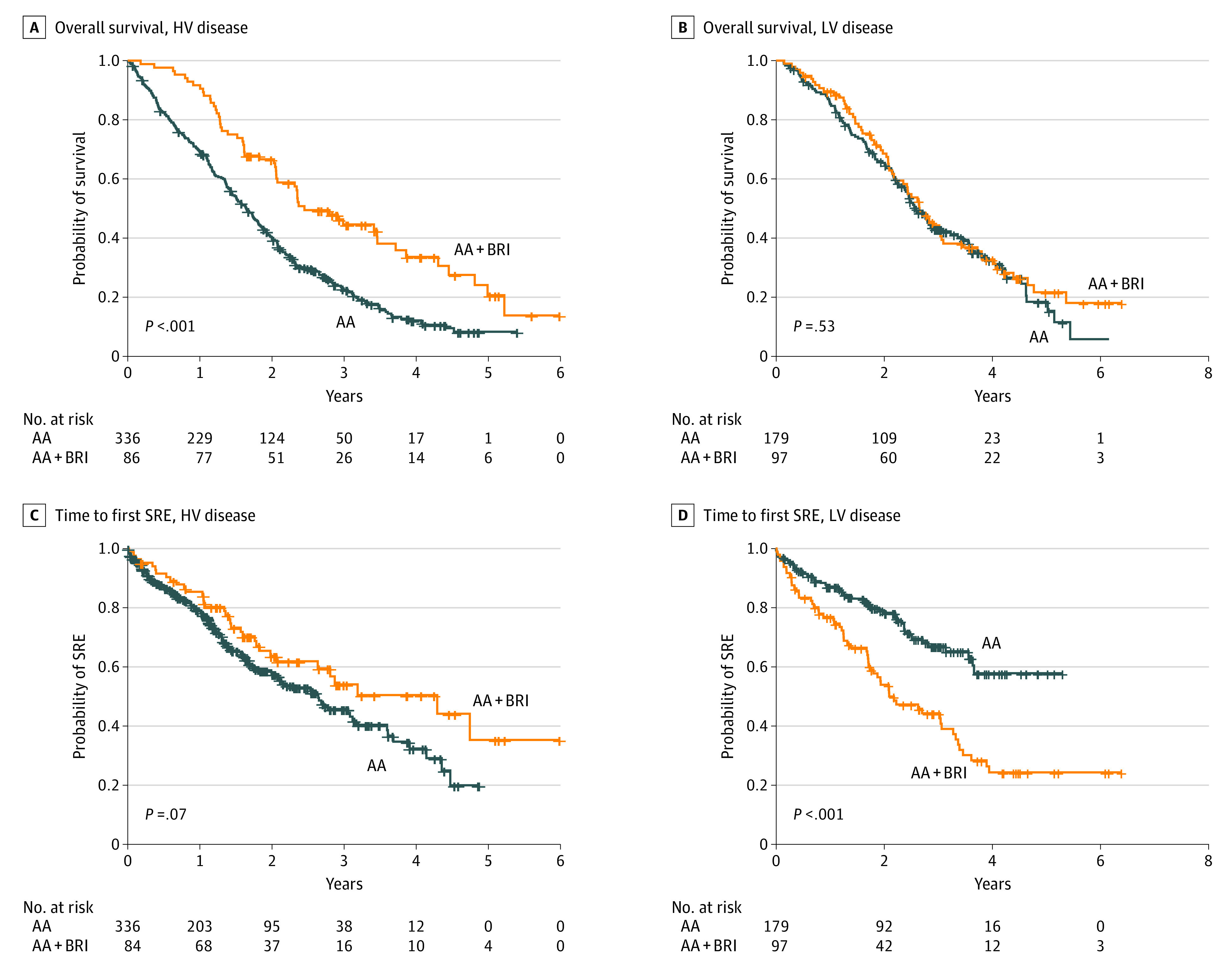
Overall Survival and Time to First Skeletal-Related Event (SRE) in Subsets of Patients With High-Volume (HV) and Low-Volume (LV) Disease by Receipt of Bone Resorption Inhibitors An SRE was defined as palliative surgery or radiotherapy to bone, pathological fractures, or spinal cord compression. High-volume disease was defined as visceral metastases and/or at least 4 bone metastases, including 1 or more metastases out of the axis and pelvis; LV disease, the absence of HV disease. A, Overall survival in patients with HV disease. B, Overall survival in patients with LV disease. C, Time to first SRE in patients with HV disease. D, Time to first SRE in patients with LV disease. AA indicates abiraterone acetate plus prednisone; BRI, bone resorption inhibitor.

The interaction tests of the abiraterone acetate and BRI cohorts by volume of disease (low vs high) revealed a differential effect in the subgroups for both OS and time to first SRE. This result suggests that the volume of disease modified the association of BRIs with the outcomes. In the subset of patients with high-volume disease, the median OS favored patients receiving concurrent BRIs (Figure 2A) who experienced a survival advantage (33.6 months; 95% CI, 24.8-46.3 months) compared with those not receiving concurrent BRIs (19.7 months; 95% CI, 17.3-21.8 months; *P* < .001) (Table 3). This OS benefit was greater than that observed in the overall population, with the risk of death among men who received treatment with abiraterone acetate with prednisone plus BRIs approximately halved compared with those receiving abiraterone acetate with prednisone alone (HR, 0.51; 95% CI, 0.38-0.68; *P* < .001).

**Table 3.** Clinical Outcomes for Disease Volume Subgroups by Receipt of Bone Resorption Inhibitors

Outcome	High-volume disease (n = 420)^a^				Low-volume disease (n = 276)^b^			
	Abiraterone acetate cohort (n = 336)	BRI cohort (n = 84)	HR (95% CI)	*P* value	Abiraterone acetate cohort (n = 179)	BRI cohort (n = 97)	HR (95% CI)	*P* value
Deaths, No.	268	53	NA	NA	114	63	NA	NA
OS, median (95% CI), mo	19.7 (17.3-21.8)	33.6 (24.8-46.3)	0.51 (0.38-0.68)	<.001	33.0 (29.2-41.4)	31.8 (28.2-37.1)	0.93 (0.68-1.26)	.62
SREs, No.	135	34	NA	NA	49	59	NA	NA
Time to first SRE, median (95% CI), mo	31.7 (25.2-38.1)	51.5 (31.6-NA)	0.70 (0.48-1.03)	.07	NA (43.9-NA)	25.2 (20.9-40.0)	2.29 (1.57-3.35)	<.001

Abbreviations: BRI, bone resorption inhibitor; HR, hazard ratio; NA, not available/not applicable; OS, overall survival; SRE, skeletal-related event.

^a^ High-volume disease was defined as visceral metastases and/or at least 4 bone metastases, including 1 or more metastases out of the axis and pelvis.

^b^ Low-volume disease was defined as the absence of high-volume disease.

In contrast, there was no difference in median OS among those with low-volume disease, regardless of whether they received concurrent BRIs (31.8 months [95% CI, 28.2-37.1 months] in the BRI cohort vs 33.0 months [95% CI, 29.2-41.4 months] in the abiraterone acetate cohort; *P* = .62) (Figure 2B). The detrimental consequences of concurrent BRI on the median time to first SRE observed in the whole population was most substantial in the subgroup with low-volume disease (Figure 2D). Patients with low-volume disease in the BRI cohort had a greater than 2-fold risk of experiencing an SRE compared with those in the abiraterone acetate cohort (HR, 2.29; 95% CI, 1.57-3.35; *P* < .001). In contrast, patients with high-volume disease in the BRI cohort experienced a delay in the median time to first SRE compared with those with high-volume disease in the abiraterone acetate cohort (HR, 0.70; 95% CI, 0.48-1.03; *P* = .07) (Figure 2C); however, this association was not statistically significant.

## Discussion

This large international cohort study is the first, to our knowledge, to evaluate the clinical impact associated with the addition of BRIs (zoledronic acid or denosumab) to abiraterone acetate with prednisone as first-line therapy for the treatment of patients with mCRPC and bone metastases. In this analysis the addition of BRIs to first-line treatment with abiraterone acetate with prednisone was associated with improvement in survival compared with abiraterone acetate with prednisone alone. This survival association is consistent with the increasing body of evidence suggesting that treatment with concurrent BRIs may provide an additive benefit to life-prolonging agents approved for the treatment of mCRPC.^14,15^ In particular, a post hoc analysis of data from the COU-AA-302 study,^14^ which assessed the clinical outcomes of concurrent BRI use among men receiving abiraterone acetate with prednisone or prednisone alone in a chemotherapy-naive setting, reported a survival benefit associated with the combination of BRIs and abiraterone acetate with prednisone compared with BRIs and prednisone alone (HR, 0.71; 95% CI, 0.50-1.00; *P* = .05). Because of the vintage of the COU-AA-302 study, most patients (93%) received treatment with zoledronic acid rather than denosumab.^14^

However, the phase 3b study conducted by the Radium-223 International Early Access Program Investigators,^15^ which examined the use of radium-223 plus concomitant anticancer agents for men with CRPC and bone metastases, suggested improved survival for those treated with concomitant denosumab (median, not available [NA]; 95% CI, 15 months to NA) compared with those who received radium-223 alone (median, 13 months; 95% CI, 12 months to NA).^15^ In the present analysis, both BRI agents were used in a more balanced distribution, with 38.9% of patients receiving zoledronic acid and 61.1% of patients receiving denosumab, and the type of BRI received was not associated with OS in the univariable and multivariable analyses, suggesting that the class of BRI may not be associated with OS findings. Therefore, a physician’s choice of type of BRI could be based on patient preference, risk-benefit scenarios, and costs.

The present study is also the first, to our knowledge, to describe the outcomes associated with the addition of BRIs to abiraterone acetate with prednisone as first-line therapy for men with CRPC and bone metastases according to disease burden. Although international guidelines recommend the use of BRIs for most men with CRPC and bone metastases with the aim of preventing SREs, the volume of disease is currently not addressed.^11,12^ Nonetheless, an increasing body of evidence suggests that the volume of disease may be associated with survival and the risk of SREs among patients with prostate cancer.^19,21,24^ In the present analysis, men with high-volume disease and worse prognosis who received concomitant BRIs had a clear survival benefit and a lower risk of experiencing a first SRE compared with those who did not receive BRIs. In contrast, in the subset of patients with low-volume disease, the receipt of concurrent BRIs was not associated with an OS advantage and, for unclear reasons, the risk of experiencing a first SRE was almost 2-fold among those in the BRI cohort compared with those in the abiraterone acetate cohort.

With respect to the time to first SRE outcomes, the data in the present study are consistent with those of a contemporary single-institution analysis performed by McGregor et al,^24^ which reported a lower risk of experiencing a first SRE among those receiving abiraterone acetate with prednisone or the androgen receptor targeting agent enzalutamide plus BRIs when used as first-line therapy compared with second-line therapy or no receipt of therapy (HR, 0.58; 95% CI, 0.36-0.95). This reduction in SRE risk was limited to those with a disease burden of 4 bone metastases or more, which approximates the E3805 definition of high-volume disease used in the present study.^19^ A post hoc analysis of the randomized phase 3 ERA 223 (Addition of Radium-223 to Abiraterone Acetate and Prednisone or Prednisolone in Patients With Castration-Resistant Prostate Cancer and Bone Metastases) study,^25^ which compared the efficacy of the combination of abiraterone acetate with prednisone plus radium-223 vs abiraterone acetate with prednisone alone, found a lower rate of bone fractures among patients receiving concomitant BRIs compared with those not receiving BRIs, regardless of the treatment arm. Of note, approximately two-thirds of the ERA 223 study population had at least 6 bone metastases.^25^ These collective findings support the hypothesis that the addition of BRIs to abiraterone acetate with prednisone may have a protective benefit for the bones of patients with high-volume disease, as observed in the present study.

In the multivariable analysis of the overall population, the volume of disease was found to be independently associated with time to first SRE. Therefore, the negative outcome associated with concurrent BRIs for time to first SRE seen in the overall population could be primarily associated with the detrimental consequences occurring in the subset of patients with low-volume disease. However, there is currently no clear biological rationale that may explain the deleterious consequences of the addition of BRIs to abiraterone acetate with prednisone therapy for time to first SRE among men with low-volume disease, and these findings should be interpreted with caution.

### Limitations

This study has limitations. Skeletal-related events, albeit defined as any palliative radiotherapy or surgery to bone metastases, pathological fractures, or spinal cord compression, were not adjudicated or categorized by type when recorded. Therefore, we have no information about how the types of SREs were distributed in each group, which could have offered insight into time to first SRE outcomes. One possibility is that palliative radiotherapy was used earlier among men with low-volume disease rather than proceeding with chemotherapy, whereas those with high-volume disease were more likely to receive treatment with chemotherapy for symptomatic progression, and these differences may have produced the observed shortening of time to first SRE in the subset of patients with low-volume disease. Thus, further prospective studies and meta-analyses are warranted to confirm these findings and acquire clearer insights on the potential differential benefits of the use of concurrent BRIs plus abiraterone acetate with prednisone for the treatment of CRPC and bone metastases among patients with high-volume vs low-volume disease and the biological mechanisms underpinning this phenomenon.

This study is also limited by its retrospective design and lack of randomization. In addition, the numerical and clinical imbalances among the cohorts may have biased the results. However, in an additional survival analysis stratified by covariates, the receipt of concomitant BRIs was associated with a survival advantage, regardless of the covariate used for stratification (eTable 4 in the Supplement). Furthermore, the survival advantage conferred by BRI receipt remained significant in the univariable and multivariable analyses of OS stratified by study center (eTable 5 in the Supplement).

Another potential limitation to this study is the limited racial diversity of the population (93.8% White). However, because the clinician’s choice was not directed by any specific protocol criteria, this study yields useful real-world data that reflect clinical practice in the Western world (eTable 6 in the Supplement). In this regard, it should be noted that most patients in our study (71.0%) did not receive BRIs in combination with first-line abiraterone acetate with prednisone therapy and, notably, most of these men (63.5%) had a high metastatic burden (Table 1). These data are similar to those reported in the study by McGregor et al,^24^ in which 50% of patients did not receive BRIs in combination with either first-line treatment with abiraterone acetate with prednisone or enzalutamide, and most of those patients (58%) had 4 or more bone metastases. This finding may reflect the common clinical behavior of withholding the use of BRIs for the treatment of CRPC with bone metastases, which is contrary to international guideline recommendations^11,12^ and may suggest physician skepticism regarding the utility of the addition of BRIs to a therapy such as abiraterone acetate with prednisone, which has known SRE-delaying and survival-improving properties.

The present study found that the addition of BRIs to abiraterone acetate with prednisone was associated with a robust survival advantage compared with abiraterone acetate with prednisone alone, and this benefit was greater among patients with high-volume disease. However, given these limitations, the results cannot be generalized and warrant further confirmation by future prospective clinical trials.

## Conclusions

In this large multicenter retrospective cohort study, the addition of BRIs to abiraterone acetate with prednisone as first-line therapy for patients with mCRPC and bone metastases was associated with significantly longer survival compared with the use of abiraterone acetate with prednisone alone. A greater survival benefit was observed among those with high-volume disease receiving concomitant BRIs. Although these findings are not generalizable, they provide real-world data in favor of the addition of BRIs to abiraterone acetate with prednisone as first-line therapy for patients with mCRPC and bone metastases, and they support the international guideline recommendation of using BRIs in this disease setting.

## Notes

Supplement. **eTable 1.** Characteristics of Patients With Complete and Incomplete Data **eTable 2.** Univariate and Multivariate Analyses of Overall Survival in Overall Population **eTable 3.** Univariate and Multivariate Analyses of Time to First Skeletal-related Event in Overall Population **eTable 4.** Overall Survival Analysis According to BRI Use Stratified by Covariates **eTable 5.** Univariable and Multivariable Analyses of Overall Survival in Overall Population Stratified by Center **eTable 6.** Patient Characteristics and Overall Survival According to BRI Use Stratified by Center **eFigure.** Study Population Flowchart

## References

[1] International Agency for Research on Center, World Health Organization. Prostate fact sheet. Global Cancer Observatory; 2020. Accessed August 30, 2020. https://gco.iarc.fr/today/data/factsheets/cancers/27-Prostate-fact-sheet.pdf

[2] Scher HI, Solo K, Valant J, Todd MB, Mehra M. Prevalence of prostate cancer clinical states and mortality in the United States: estimates using a dynamic progression model. PLoS One. 2015;10(10):e0139440. doi:10.1371/journal.pone.0139440 26460686 PMC4603789

[3] Ryan CJ, Smith MR, de Bono JS, ; COU-AA-302 Investigators. Abiraterone in metastatic prostate cancer without previous chemotherapy. N Engl J Med. 2013;368(2):138-148. doi:10.1056/NEJMoa1209096 23228172 PMC3683570

[4] Beer TM, Armstrong AJ, Rathkopf DE, ; PREVAIL Investigators. Enzalutamide in metastatic prostate cancer before chemotherapy. N Engl J Med. 2014;371(5):424-433. doi:10.1056/NEJMoa1405095 24881730 PMC4418931

[5] Sathiakumar N, Delzell E, Morrisey MA, . Mortality following bone metastasis and skeletal-related events among men with prostate cancer: a population-based analysis of US Medicare beneficiaries, 1999-2006. Prostate Cancer Prostatic Dis. 2011;14(2):177-183. doi:10.1038/pcan.2011.7 21403668

[6] Oudard S, Banu E, Medioni J, . What is the real impact of bone pain on survival in patients with metastatic hormone-refractory prostate cancer treated with docetaxel? BJU Int. 2009;103(12):1641-1646. doi:10.1111/j.1464-410X.2008.08283.x 19210673

[7] Berruti A, Tucci M, Mosca A, . Predictive factors for skeletal complications in hormone-refractory prostate cancer patients with metastatic bone disease. Br J Cancer. 2005;93(6):633-638. doi:10.1038/sj.bjc.6602767 16222309 PMC2361623

[8] Saad F, Gleason DM, Murray R, ; Zoledronic Acid Prostate Cancer Study Group. A randomized, placebo-controlled trial of zoledronic acid in patients with hormone-refractory metastatic prostate carcinoma. J Natl Cancer Inst. 2002;94(19):1458-1468. doi:10.1093/jnci/94.19.1458 12359855

[9] Saad F, Gleason DM, Murray R, ; Zoledronic Acid Prostate Cancer Study Group. Long-term efficacy of zoledronic acid for the prevention of skeletal complications in patients with metastatic hormone-refractory prostate cancer. J Natl Cancer Inst. 2004;96(11):879-882. doi:10.1093/jnci/djh141 15173273

[10] Fizazi K, Carducci M, Smith M, . Denosumab versus zoledronic acid for treatment of bone metastases in men with castration-resistant prostate cancer: a randomised, double-blind study. Lancet. 2011;377(9768):813-822. doi:10.1016/S0140-6736(10)62344-6 21353695 PMC3090685

[11] Mohler JL, Antonarakis ES, Armstrong AJ, . Prostate cancer, version 2.2019, NCCN clinical practice guidelines in oncology. J Natl Compr Canc Netw. 2019;17(5):479-505. doi:10.6004/jnccn.2019.0023 31085757

[12] Cornford P, Bellmunt J, Bolla M, . EAU-ESTRO-SIOG guidelines on prostate cancer. part II: treatment of relapsing, metastatic, and castration-resistant prostate cancer. Eur Urol. 2017;71(4):630-642. doi:10.1016/j.eururo.2016.08.002 27591931

[13] Saad F, Sternberg CN, Mulders PFA, Niepel D, Tombal BF. The role of bisphosphonates or denosumab in light of the availability of new therapies for prostate cancer. Cancer Treat Rev. 2018;68:25-37. doi:10.1016/j.ctrv.2018.04.014 29787892

[14] Saad F, Shore N, Van Poppel H, . Impact of bone-targeted therapies in chemotherapy-naive metastatic castration-resistant prostate cancer patients treated with abiraterone acetate: post hoc analysis of study COU-AA-302. Eur Urol. 2015;68(4):570-577. doi:10.1016/j.eururo.2015.04.032 25985882 PMC5056561

[15] Saad F, Carles J, Gillessen S, ; Radium-223 International Early Access Program Investigators. Radium-223 and concomitant therapies in patients with metastatic castration-resistant prostate cancer: an international, early access, open-label, single-arm phase 3b trial. Lancet Oncol. 2016;17(9):1306-1316. doi:10.1016/S1470-2045(16)30173-5 27473888

[16] Logothetis CJ, Basch E, Molina A, . Effect of abiraterone acetate and prednisone compared with placebo and prednisone on pain control and skeletal-related events in patients with metastatic castration-resistant prostate cancer: exploratory analysis of data from the COU-AA-301 randomised trial. Lancet Oncol. 2012;13(12):1210-1217. doi:10.1016/S1470-2045(12)70473-4 23142059

[17] Flaig TW, Potluri RC, Ng Y, Todd MB, Mehra M. Treatment evolution for metastatic castration-resistant prostate cancer with recent introduction of novel agents: retrospective analysis of real-world data. Cancer Med. 2016;5(2):182-191. doi:10.1002/cam4.576 26710718 PMC4735776

[18] Iuliani M, Pantano F, Buttigliero C, . Biological and clinical effects of abiraterone on anti-resorptive and anabolic activity in bone microenvironment. Oncotarget. 2015;6(14):12520-12528. doi:10.18632/oncotarget.3724 25904051 PMC4494955

[19] Sweeney CJ, Chen YH, Carducci M, . Chemohormonal therapy in metastatic hormone-sensitive prostate cancer. N Engl J Med. 2015;373(8):737-746. doi:10.1056/NEJMoa1503747 26244877 PMC4562797

[20] Scher HI, Morris MJ, Stadler WM, ; Prostate Cancer Clinical Trials Working Group 3. Trial design and objectives for castration-resistant prostate cancer: updated recommendations from the Prostate Cancer Clinical Trials Working Group 3. J Clin Oncol. 2016;34(12):1402-1418. doi:10.1200/JCO.2015.64.2702 26903579 PMC4872347

[21] Francini E, Gray KP, Xie W, . Time of metastatic disease presentation and volume of disease are prognostic for metastatic hormone sensitive prostate cancer (mHSPC). Prostate. 2018;78(12):889-895. doi:10.1002/pros.23645 29707790 PMC6171350

[22] Miller K, Carles J, Gschwend JE, Van Poppel H, Diels J, Brookman-May SD. The phase 3 COU-AA-302 study of abiraterone acetate plus prednisone in men with chemotherapy-naive metastatic castration-resistant prostate cancer: stratified analysis based on pain, prostate-specific antigen, and Gleason score. Eur Urol. 2018;74(1):17-23. doi:10.1016/j.eururo.2017.08.035 28939004

[23] Pond GR, Sonpavde G, de Wit R, Eisenberger MA, Tannock IF, Armstrong AJ. The prognostic importance of metastatic site in men with metastatic castration-resistant prostate cancer. Eur Urol. 2014;65(1):3-6. doi:10.1016/j.eururo.2013.09.024 24120464

[24] McGregor B, Zhang L, Gray KP, . Bone targeted therapy and skeletal related events in the era of enzalutamide and abiraterone acetate for castration resistant prostate cancer with bone metastases. Prostate Cancer Prostatic Dis. 2021;24(2):341-348. doi:10.1038/s41391-020-00280-6 32884090

[25] Smith M, Parker C, Saad F, . Addition of radium-223 to abiraterone acetate and prednisone or prednisolone in patients with castration-resistant prostate cancer and bone metastases (ERA 223): a randomised, double-blind, placebo-controlled, phase 3 trial. Lancet Oncol. 2019;20(3):408-419. doi:10.1016/S1470-2045(18)30860-X 30738780

