# Supplementary material for: Association of Concomitant Bone Resorption Inhibitors With Overall Survival Among Patients With Metastatic Castration-Resistant Prostate Cancer and Bone Metastases Receiving Abiraterone Acetate With Prednisone as First-Line Therapy
Source: JAMA Netw Open. 2021 Jul 22;4(7):e2116536. doi: 10.1001/jamanetworkopen.2021.16536 (PMC8299314; doi:10.1001/jamanetworkopen.2021.16536)
Supplement: Supplement. — eTable 1. Characteristics of Patients With Complete and Incomplete Data eTable 2. Univariate and Multivariate Analyses of Overall Survival in Overall Population eTable 3. Univariate and Multivariate Analyses of Time to First Skeletal-related Event in Overall Population eTable 4. Overall Survival Analysis According to BRI Use Stratified by Covariates eTable 5. Univariable and Multivariable Analyses of Overall Survival in Overall Population Stratified by Center eTable 6. Patient Characteristics and Overall Survival According to BRI Use Stratified by Center eFigure. Study Population Flowchart [file jamanetwopen-e2116536-s001.pdf]

Supplementary Online Content

Francini E, Montagnani F, Nuzzo PV, et al. Association of concomitant bone resorption inhibitors with overall survival among patients with metastatic castration-resistant prostate cancer and bone metastases receiving abiraterone acetate with prednisone as first-line therapy. *JAMA Netw Open*. 2021;4(7):e2116536. doi:10.1001/jamanetworkopen.2021.16536

eTable 1. Characteristics of Patients With Complete and Incomplete Data

eTable 2. Univariate and Multivariate Analyses of Overall Survival in Overall Population

eTable 3. Univariate and Multivariate Analyses of Time to First Skeletal-related Event in Overall Population

eTable 4. Overall Survival Analysis According to BRI Use Stratified by Covariates

eTable 5. Univariable and Multivariable Analyses of Overall Survival in Overall Population Stratified by Center

eTable 6. Patient Characteristics and Overall Survival According to BRI Use Stratified by Center

eFigure. Study Population Flowchart

This supplementary material has been provided by the authors to give readers additional information about their work.

© 2021 Francini E et al. *JAMA Network Open*.

eTable 1. Characteristics of Patients With Complete and Incomplete Data

Variable	Overall population n=745	Patients with available data n=678	*P* value
Median age at baseline, yrs (IQR)	77.6 (68.1-83.6)	77.3 (67.8-83.1)	0.96
Race, No. (%)			0.99
Caucasian	699 (93.8)	635 (93.6)	
Other	19 (2.5)	18 (2.7)	
Not available	27 (3.7)	25 (3.6)	
Gleason score, No. (%)			0.98
≤6	62 (8.3)	57 (8.4)	
7	218 (29.3)	200 (29.5)	
≥8	337 (45.2)	310 (45.7)	
Not available	128 (17.2)	111 (16.4)	
Prior local therapy, No. (%)			0.93
No	353 (47.4)	328 (48.4)	
Surgery	196 (26.3)	177 (26.1)	
Radiotherapy	181 (24.3)	173 (25.5)	
Not available	15 (2.0)	0	
De novo, No. (%)			0.70
No	483 (64.9)	433 (63.9)	
Yes	262 (35.1)	245 (36.1)	
Treatment for mHSPC, No. (%)			0.82
ADT	617 (82.8)	602 (88.8)	
ADT+D	81 (10.8)	76 (11.2)	
Not available	47 (6.4)	0	
Volume of disease at baseline, No. (%)			0.99
Low	276 (37.0)	269 (39.7)	
High	420 (56.4)	409 (60.3)	
Not available	49 (6.6)	0	
Visceral metastases at baseline, No. (%)			0.99
No	680 (91.3)	619 (91.3)	
Yes	65 (8.7)	59 (8.7)	
Liver metastases at baseline, No. (%)			0.62
No	709 (95.2)	649 (95.7)	
Yes	36 (4.8)	29 (4.3)	
ECOG PS at baseline, No. (%)			0.55
0	324 (43.5)	316 (46.7)	
≥1	369 (49.5)	362 (53.3)	
Not available	52 (7.0)	0	
Pain intensity at baseline, No. (%)			0.94
VAS 0-3	514 (69.0)	506 (74.6)	
VAS 4-5	112 (15.0)	107 (15.8)	
VAS >5	70 (9.4)	65 (9.6)	
Not available	49 (6.6)	0	
Use of opioids at baseline, No. (%)			0.79
No	598 (80.3)	585 (86.3)	
Yes	99 (13.3)	93 (13.7)	
Not available	48 (6.4)	0	
Median PSA at baseline, ng/mL (IQR)	32 (10.0-98.5)	32.3 (10.2 – 99.7)	0.95
Use of BRI, No. (%)			0.36
No	529 (71)	504 (74.3)	

© 2021 Francini E et al. *JAMA Network Open.*

Zoledronic Acid<br>Denosumab	84 (11.3)<br>132 (17.7)	70 (11.3)<br>104 (14.2)	
Median time from start of AA to start of BRI, days (IQR)	15 (0-68)	15 (0-67)	0.99
Median time of BRI administration, days (IQR)	741 (362-1182)	738 (357-1174)	0.94
Median AA cycles, n (IQR)	9 (4-20)	9 (4-20)	0.99
Pain palliation, No. (%)<br>No<br>Yes<br>Not available	 198 (26.6)<br>497 (66.7)<br>50 (6.7)	 190 (28)<br>486 (71.7)<br>2 (0.3)	0.87
Number of treatments received after AA, Median (IQR)<br>0-1<br>>1<br>Not available	1 (0-2)<br>511 (68.6)<br>225 (30.2)<br>9 (1.2)	1 (0-2)<br>488 (72)<br>190 (28)<br>0	0.99<br><br>0.29
Median FU in overall population, months (CI)	23.5 (19.8-24.9)	23.5 (19.7 – 24.8)	0.94

Abbreviations: AA = abiraterone acetate plus prednisone; ADT = androgen deprivation therapy; BRI = bone resorption inhibitors; CI = confidence interval; D = docetaxel; ECOG PS = Eastern Cooperative Oncology Group performance status; FU = follow-up; IQR = interquartile range; mHSPC = metastatic hormone-sensitive prostate cancer; NA = not available; PSA = prostate-specific antigen; VAS = visual analogue scale; yrs = years.
Note: High volume is defined as ≥4 bone metastases of which at least 1 out of the pelvis and axis and/or visceral metastases); low volume is defined as not high volume.

© 2021 Francini E et al. *JAMA Network Open*.

eTable 2. Univariate and Multivariate Analyses of Overall Survival in Overall Population

**Variable**	**Univariable Analysis**		**Multivariable Analysis**	
	**HR (95%CI)**	***P* value**	**HR (95%CI)**	***P* value**
Gleason grade (ref. ≤6)			NA	NA
7	0.82 (0.58-1.16)	.26		
≥8	1.06 (0.76-1.49)	.72		
Prior local therapy (ref. no)				
Surgery	0.62 (0.50-0.78)	<.001	0.51 (0.39-0.68)	<.001
Radiotherapy	0.73 (0.59-0.91)	.005	0.71 (0.55-0.93)	.03
De-novo (ref. no)	1.34 (1.12-1.61)	.001	NS	NS
Treatment for mHSPC (ref. ADT alone)	0.97 (0.74-1.29)	.87	NA	NA
Volume of disease at baseline (ref. high volume)	0.60 (0.50-0.72)	<.001	0.66 (0.54-0.80)	<.001
Visceral metastases at baseline (ref. no)	1.45 (1.08-1.95)	.01	NS	NS
Liver metastases at baseline (ref. no)	1.75 (1.20-2.54)	.004	2.04 (1.37-3.05)	.006
ECOG PS at baseline (ref. 0)	1.78 (1.49-2.13)	<.001	1.46 (1.20-1.78)	.003
Pain intensity at baseline (ref. VAS 0-3)				
4-5	1.26 (1.00-1.59)	.057	1.13 (0.87-1.45)	.35
>5	2.03 (1.54-2.66)	<.001	1.88 (1.39-2.54)	<.001
Use of opioids at baseline (ref. no)	1.64 (1.30-2.08)	<.001	NS	NS
Use of BRI (ref. no)	0.65 (0.54-0.79)	<.001	0.64 (0.52-0.79)	<.001
PSA at baseline (as a continuous variable)	1.05 (1.03-1.06)	<.001	1.03 (1.01-1.05)	.006
Type of BRI used (ref. zoledronic acid)	0.93 (0.66 – 1.30)	.79	NA	NA

Abbreviations: ADT = androgen deprivation therapy; BRI = bone resorption inhibitors; CI = confidence interval; ECOG PS = Eastern Cooperative Oncology Group performance status; mHSPC = metastatic hormone-sensitive prostate cancer; HR = hazard ratio; NA = not available; NS = not significant; PSA = prostate-specific antigen; ref. = reference; VAS = visual analogue scale
Note: the multivariable analysis included a subset of patients (n=678) with all available covariates data.
High volume is defined as ≥4 bone metastases of which at least 1 out of the pelvis and axis and/or visceral metastases); low volume is defined as not high volume.

© 2021 Francini E et al. *JAMA Network Open.*

eTable 3. Univariate and Multivariate Analyses of Time to First Skeletal-related Event in Overall Population

Variable	Univariable Analysis HR (95%CI)	*P* value	Multivariable Analysis HR (95%CI)	*P* value
Gleason grade (ref. ≤6)<br>7<br>≥8	 <br>1.03 (0.65-1.63)<br>1.13 (0.72-1.75)	 <br>.88<br>.59	NA	NA
Prior local therapy (ref. no)<br>Surgery<br>Radiotherapy	 <br>0.79 (0.60-1.03)<br>0.75 (0.56-1.01)	 <br>.08<br>.05	NA	NA
De-novo (ref. no)	1.43 (1.13-1.81)	.002	NA	NA
Treatment for mHSPC (ref. ADT alone)	1.02 (0.71-1.47)	.90	NA	NA
Volume of disease at baseline (ref. high volume)	0.74 (0.58-0.95)	.02	0.73 (0.57-0.93)	.01
Visceral metastases at baseline (ref. no)	0.99 (0.63-1.56)	.96	NA	NA
Liver metastases at baseline (ref. no)	1.43 (0.83-2.44)	.19	NA	NA
ECOG PS at baseline (ref. 0)	1.53 (1.21-1.94)	.005	NS	NS
Pain intensity at baseline (ref. VAS 0-3)<br>4-5<br>>5	 <br>1.51 (1.10-2.05)<br>3.42 (2.43-4.82)	 <br>.009<br><.001	 <br>1.50 (1.10-2.04) 3.52<br>(2.49-4.98)	 <br>.001<br><.001
Use of opioids at baseline (ref. no)	2.42 (1.81-3.23)	<.001	NS	NS
Use of BRI (ref. no)	1.27 (1.00-1.60)	.04	NS	NS
PSA at baseline (as a continuous variable)	1.02 (0.98-1.05)	.08	NA	NA

Abbreviations: ADT = androgen deprivation therapy; BRI = bone resorption inhibitors; CI = confidence interval; ECOG PS = Eastern Cooperative Oncology Group performance status; mHSPC = metastatic hormone-sensitive prostate cancer; HR = hazard ratio; NA = not available; NS = not significant; PSA = prostate-specific antigen; ref. = reference; VAS = visual analogue scale
Note: the multivariable analysis included a subset of patients (n=685) with all available covariates data.
High volume is defined as ≥4 bone metastases of which at least 1 out of the pelvis and axis and/or visceral metastases); low volume is defined as not high volume.

© 2021 Francini E et al. *JAMA Network Open*.

eTable 4. Overall Survival Analysis According to BRI Use Stratified by Covariates

**Variables used for stratification**	**HR (95% CI)**	***P* value**
Center	0.75 (0.61-0.93)	.01
Volume of disease at baseline (ref. high volume)	0.67 (0.54-0.82)	.002
Prior local therapy (ref. no)	0.68 (0.56-0.83)	.001
De-novo (ref. no)	0.65 (0.54-0.78)	<.001
Liver metastases at baseline (ref. no)	0.65 (0.53-0.79)	<.001
Visceral metastases at baseline (ref. no)	0.66 (0.54-0.80)	<.001
ECOG PS at baseline (ref. 0)	0.66 (0.54-0.82)	.001
Pain intensity at baseline (ref. VAS 0-3)	0.61 (0.49-0.75)	<.001
Use of opioid at baseline (ref. no)	0.59 (0.48-0.73)	<.001
PSA at baseline (> median value)	0.66 (0.55-0.80)	<.001

Abbreviations: BRI = bone resorption inhibitors; CI = confidence interval; ECOG PS = Eastern Cooperative Oncology Group performance status; HR = hazard ratio; PSA = prostate-specific antigen
Note: Median PSA = 32 ng/ml. High volume is defined as ≥4 bone metastases of which at least 1 out of the pelvis and axis and/or visceral metastases); low volume is defined as not high volume.

© 2021 Francini E et al. *JAMA Network Open*.

eTable 5. Univariable and Multivariable Analyses of Overall Survival in Overall Population Stratified by Center

Variable	Univariable Analysis HR (95%CI)	Univariable Analysis *P* value	Multivariable Analysis HR (95%CI)	Multivariable Analysis *P* value
Gleason grade (ref. ≤6)				
7	0.82 (0.58-1.16)	0.26	NA	NA
≥8	1.06 (0.76-1.49)	0.72		
Prior local therapy (ref. no)				
Surgery	0.62 (0.50-0.78)	<.001	0.58 (0.45-0.73)	<.001
Radiotherapy	0.73 (0.59-0.91)	.005	0.79 (0.63-0.99)	.04
De-novo (ref. no)	1.34 (1.12-1.60)	.001	NS	NS
Treatment for mHSPC (ref. ADT alone)	1.26 (0.93-1.71)	.14	NA	NA
Volume of disease at baseline (ref. high volume)	0.72 (0.58-0.88)	.001	0.68 (0.55-0.84)	.004
Visceral metastases at baseline (ref. no)	1.43 (1.06-1.94)	.02	NS	NS
Liver metastases at baseline (ref. no)	1.96 (1.33-2.89)	.007	1.97 (1.29-3.01)	.002
ECOG PS at baseline (ref. 0)	1.62 (1.33-1.97)	<.001	1.42 (1.16-1.75)	.008
Pain intensity at baseline (ref. VAS 0-3)				
4-5	1.43 (1.10-1.84)	.006	1.14 (0.87-1.49)	0.33
>5	1.96 (1.47-2.60)	<.001	1.86 (1.37-2.52)	<.001
Use of opioids at baseline (ref. no)	1.80 (1.41-2.86)	<.001	NS	NS
Use of BRI (ref. no)	0.75 (0.61-0.93)	0.01	0.67 (0.51-0.88)	.004
PSA at baseline (as a continuous variable)	1.06 (1.05-1.07)	<.001	1.03 (1.01-1.04)	.001
Type of BRI used (ref. zoledronic acid)	0.97 (0.69-1.35)	.81	NA	NA

Abbreviations: ADT = androgen deprivation therapy; BRI = bone resorption inhibitors; CI = confidence interval; ECOG PS = Eastern Cooperative Oncology Group performance status; mHSPC = metastatic hormone-sensitive prostate cancer; HR = hazard ratio; NA = not available; NS = not significant; PSA = prostate-specific antigen; ref. = reference; VAS = visual analogue scale
Note: the multivariable analysis included a subset of patients (n=678) with all available covariates data. High volume is defined as ≥4 bone metastases of which at least 1 out of the pelvis and axis and/or visceral metastases); low volume is defined as not high volume.

© 2021 Francini E et al. *JAMA Network Open*.

eTable 6. Patient Characteristics and Overall Survival According to BRI Use Stratified by Center

Center	Number of Patients	Median OS, months (95% CI)	% BRI administration	Mean subsequent treatments	% ECOG PS >1	% high volume	Median baseline PSA (IQR)
1	24	26.8 (18.0-NR)	20.8	0.6	45.8	66.7	67 (11.7-116.3)
2	39	29.3 (24.5-NR)	80.0	1.2	63.1	24.2	8.0 (3.5-36.0)
3	26	37.4 (21.6-NR)	11.5	0.8	46.1	46.1	15.5 (4.25-51.5)
4	26	24.4 (16.4-NR)	19.2	0.8	30.8	30.8	12.0 (4.0-39.3)
5	126	36.5 (33.4-50.1)	47.2	2.3	15.1	33.3	16.1 (3.4-45.2)
6	393	21 (18.7-23.5)	6.9	0.6	64.8	80.0	55.6 (21.2-155.2)
7	10	34.8 (16.7-NR)	70.0	1.2	40.0	50.0	24.9 (15.3-65.1)
8	45	33.1 (22.8-49.2)	73.0	3.3	NA*	NA*	15.4 (7.62-58.5)

Abbreviations: BRI = bone resorption inhibitors; CI = confidence interval; ECOG PS = Eastern Cooperative Oncology Group performance status; HR = hazard ratio; IQR = interquartile range; NA = not available; NR = not reached; PSA = prostate-specific antigen

Note: High volume is defined as ≥4 bone metastases of which at least 1 out of the pelvis and axis and/or visceral metastases); low volume is defined as not high volume.

* data for ECOG-PS and volume were not available for this center.

© 2021 Francini E et al. *JAMA Network Open*.

eFigure. Study Population Flowchart

eFigure 1. Study population flow.

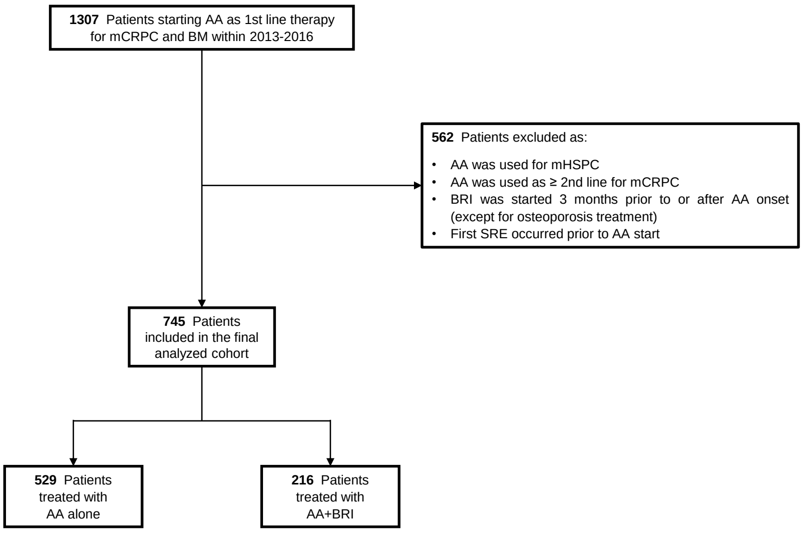

Legend: AA = abiraterone acetate plus prednisone; BM = bone metastases; BRI = bone-resorption inhibitors (zoledronic acid or denosumab); mCRPC = metastatic castration-resistant prostate cancer; mHSPC = metastatic hormone-sensitive prostate cancer; SRE = skeletal-related event (definition: palliative radiotherapy or surgery to bone, pathological fractures, or spinal cord compression)
Note: Number of deaths prior to data cut-off date (September 15, 2019) were 369 among those treated with AA and 147 among those treated with AA+BRI.

© 2021 Francini E et al. *JAMA Network Open*.
